# A New Mesoscopic Parameter Inverse Analysis Method of Hydraulic Concrete Based on the SVR-HGWO Intelligent Algorithm

**DOI:** 10.3390/ma18184274

**Published:** 2025-09-12

**Authors:** Qingshuai Zhu, Yuling Wang, Xing Li

**Affiliations:** 1 China South-to-North Water Diversion Corporation Limited, Building 9, East Zone, No. 9 Yard, Linglong Road, Beijing 100036, China; 2 China Power Construction Ecological Environment Group Corporation Limited, Haina Baichuan Plaza, No. 6 Baoxing Road, Haibin Community, Xinan Subdistrict, Bao’an District, Shenzhen 518101, China; 3 Nanjing Hydraulic Research Institute, No. 225, Guangzhou Road, Nanjing 210029, China

**Keywords:** mesoscopic parameter inversion, SVR, HGWO, 3D mesoscopic numerical simulation, hydraulic concrete

## Abstract

Accurate identification of mesoscopic parameters is critical for understanding the cracking and failure mechanisms of hydraulic concrete and for improving the reliability of numerical simulations. Traditional trial-and-error methods for parameter calibration are inefficient and often lack robustness. To address this issue, this study proposes a novel inversion method combining Support Vector Regression (SVR) with a Hybrid Grey Wolf Optimization (HGWO) algorithm. First, a mesoscopic simulation dataset of three-point bending (TPB) tests was constructed using 3D numerical models with varying mesoscopic parameters. Then, an SVR-based surrogate model was trained to learn the nonlinear mapping between mesoscopic parameters and load–CMOD (Crack Mouth Opening Displacement) curves. The HGWO algorithm was employed to optimize the SVR hyperparameters (penalty factor *C* and kernel coefficient *g*) and subsequently used to invert the mesoscopic parameters by minimizing the discrepancy between experimental and predicted CMOD values. The proposed method was validated through inversion of the mortar parameters of a tertiary hydraulic concrete beam. The results demonstrate that the HGWO-SVR model achieves high prediction accuracy (R^2^ = 0.944, MAE = 1.220, MAPE = 0.041) and significantly improves computational efficiency compared to traditional methods. The simulation based on the inversed parameters yields load–CMOD curves that agree well with experimental results. This approach provides a promising and efficient tool for mesoscopic parameter identification of heterogeneous materials in hydraulic structures.

## 1. Introduction

Cracking is one of the most common and typical defects in hydraulic concrete structures such as dams. The cracking mechanism of concrete, analytical theories, and methodological studies have long attracted attention in both engineering and academia. After decades of research, progress has been made in mechanical testing, numerical simulation, and failure criteria [1]. However, traditional investigations mainly relied on macroscopic experiments, which provide limited insight into underlying mechanisms. The mesoscopic behavior of hydraulic concrete, governed by the heterogeneous distribution of aggregates, mortar, and interfacial transition zones (ITZ), remains insufficiently understood [2]. Compared with macroscopic approaches, mesoscale numerical modeling enables mechanism-based prediction of fracture behavior and supports the development of effective control strategies [3,4]. Nevertheless, reliable application requires accurate identification of mesoscopic model parameters.

Earlier studies attempted to determine parameters of each concrete phase through direct experiments and trial calculations. Caballero et al. [5] and Jiaming et al. [6] obtained mortar parameters by preparing specimens without aggregates and performing mechanical tests, while ITZ parameters were inferred through trial analyses. Afnan et al. [7] analyzed large datasets of mortar specimens and established empirical relationships among tensile strength, compressive strength, the elastic modulus, and the water–cement ratio. Junfeng [8] further investigated statistical distributions of tensile strength and elastic modulus for aggregates, mortar, and ITZ. These results provided valuable insights but were highly sensitive to mix proportion, curing conditions, and environmental effects, thus lacking robustness and transferability.

More recent work has emphasized 3D mesoscale simulations, using techniques such as Voronoi tessellation or X-ray CT reconstruction to capture aggregate geometry and crack evolution with high fidelity [3,4]. For example, Huang et al. developed an efficient framework to generate realistic 3D mesoscale concrete models from micro X-ray CT images combined with a dynamic physics engine [9]. A broader review of finite element modeling of concrete meso-structures was recently provided by Pan et al. (2025), summarizing state-of-the-art approaches and challenges [10]. These advances highlight the potential of mesoscopic modeling as a “virtual laboratory” for investigating hydraulic concrete performance. Yet, parameter inversion for such models remains challenging due to computational demands, particularly in 3D.

Various parameter inversion methods have been proposed. Xu et al. [11] introduced a multi-objective inverse approach for cohesive zone models to simulate mixed-mode crack propagation. Zimmermann et al. [12] developed a stochastic inverse analysis to determine distributions of fracture parameters. Gajewski et al. [13] and Shen & Paulino [14] employed digital image correlation (DIC)-assisted modeling to calibrate fracture parameters by comparing simulated and experimental deformation fields. Ferreira et al. [15] extended such approaches to notched beams. These methods improved accuracy but were mainly limited to 2D mesoscopic models and remained computationally expensive, especially for 3D simulations.

With the rapid development of artificial intelligence, data-driven inversion methods have emerged. Machine learning models, especially support vector regression (SVR) [16], have shown promise as surrogate models that approximate nonlinear mappings between mesoscopic parameters and macroscopic responses. Recent studies have highlighted the potential of hybrid SVR approaches integrated with advanced metaheuristic algorithms, such as Harris Hawks Optimization (HHO) [17], Grey Wolf Optimizer and Firefly Algorithm [18], and other hybrid strategies. Furthermore, AI-enhanced computational mechanics has been increasingly recognized as a transformative tool in geotechnical engineering [19]. In the context of concrete materials, Nafees et al. demonstrated the effectiveness of machine learning models in predicting mechanical properties of silica fume-based green concrete [20]. These methods reduce computational burden while maintaining predictive power, providing a promising pathway for parameter inversion in hydraulic concrete.

Despite these advances, robust inversion of 3D mesoscopic parameters for hydraulic concrete is still limited, and the influence of environmental factors critical for dam safety remains underexplored. Therefore, this study proposes a novel SVR–HGWO-based framework for mesoscopic parameter inversion. A database of three-point bending (TPB) simulations is used to train the SVR model, while HGWO optimizes its hyperparameters and performs inversion against experimental results. The framework significantly improves accuracy and efficiency, providing a promising pathway toward intelligent safety assessment of hydraulic concrete structures.

## 2. Mapping Relationship Between Macro Response and Mesoscale Parameters of Hydraulic Concrete Based on SVR

**Figure 1:**
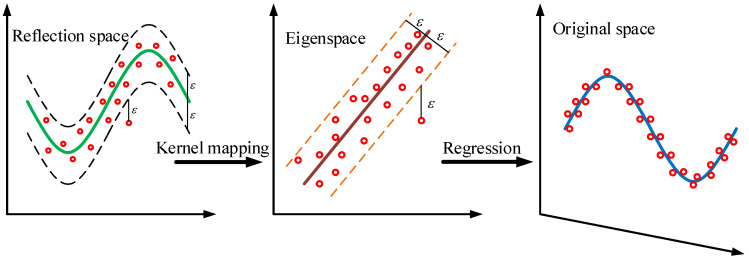
The basic principle of SVR [16].

Vapnik [16] proposed the support vector machine (SVM) theory in the 1990s based on the principle of structural risk minimization and applied the idea of SVM to solve classification and regression problems, thus obtaining the SVR model. As shown in Figure 1, the basic theory of SVR is to map the original data *x* into a feature space ***F*** with higher dimensionality through a nonlinear function *ϕ* and then perform linear regression in the feature space [21]. SVR is built on statistical learning philosophy. The application of SVR improves the anti-interference ability of the traditional model, expands its application scope, and obtains relatively ideal prediction results.

Let {(*x_i_*, *y_i_*), *i* = 1, 2, …, *n*} be a training set containing *n* training samples, where *x_i_* is the input column vector of the *i-*th training sample, and *y_i_* is the corresponding one-dimensional output value. According to the classical support vector regression (SVR) theory introduced by Vapnik et al. [16], the goal of SVR is to find a nonlinear mapping *ϕ*(*x*) from a low-dimensional nonlinear space to a high-dimensional linear space, such that

(1) y=f(x)=ωϕ(x)+b

By introducing slack variables *ξ_i_* and ξ_i_^*^, the values of regression coefficients *w* and *b* are determined by the minimum value of the following formula for linear problems:

(2) $$\underset{w,b,\xi ,\xi^{*},\varepsilon }{\min}\frac{1}{2}ww^{T}+c\left(v\varepsilon +\frac{1}{l}\sum_{i=1}^{l}\left(\xi_{i}+\xi_{i}^{*}\right)\right)$$

(3) $$\mathrm{subject}\;\mathrm{to}:\left\{\begin{array}{c} \left(w^{T}\phi (x_{i})+b\right)-y_{i}\leq \varepsilon +\xi_{i}\\ y_{i}-\left(w^{T}\phi (x_{i})+b\right)\leq \varepsilon +\xi_{i}^{*}\\ \xi_{i}\geq 0,\xi_{i}^{*}\geq 0 \end{array}\right.$$

where *c* is the penalty factor, which is a constant greater than zero, indicating the degree of penalty for training errors; *ε* is the insensitive loss coefficient; *v* ∈ [0, 1] is the insensitive loss coefficient weight factor; *ξ_i_*, ξ_i_^*^ are relaxation factors.

The optimization problem is ultimately reduced to the following quadratic programming problem by duality theory:

(4) $$\underset{\alpha ,\alpha^{*}}{\min}\frac{1}{2}(\alpha -\alpha^{*})Q(\alpha -\alpha^{*})+y^{T}(\alpha -\alpha^{*})$$

(5) $$\mathrm{subject}\;\mathrm{to}:\left\{\begin{array}{c} e^{T}(\alpha -\alpha^{*})=0,e^{T}(\alpha +\alpha^{*})\leq cv\\ \alpha_{i}\geq 0,\alpha_{i}^{*}\leq c/l \end{array}\right.$$

For linear problems, the support vector fitting function can be obtained by solving it as

(6) $$f(x)=\sum_{i=1}^{n}(\alpha_{i}^{*}-\alpha_{i})(x_{i}\cdot x)+b$$

For nonlinear regression, first, use a nonlinear mapping to map the data to a high-dimensional feature space, and then perform linear regression in the high-dimensional feature space to obtain the regression equation

(7) $$f(x)=\sum_{i=1}^{n}(\alpha_{i}^{*}-\alpha_{i})K(x_{i}\cdot x)+b$$

In the formula: *K*(*x_i_*∙ *x_j_*) = *ϕ*(*x_i_*)*ϕ*(*x_j_*) is the kernel function; α_i_^*^, *α_i_* are the multipliers corresponding to the constraints. Only a tiny part is not zero; the corresponding samples are the support vectors.

The loss function is a measure to evaluate the accuracy of SVR prediction, and its common forms are [22]

① *ε* insensitive loss function:

(8) $$L_{\varepsilon }(y-f(x))=\left\{\begin{array}{c} 0,\;\left|y-f(x)\right|\leq \varepsilon \\ \left|y-f(x)\right|-\varepsilon ,\;else \end{array}\right.$$

② Quadratic *ε* insensitive loss function:

(9) $$L_{s}(y-f(x))={\left|y-f(x)\right|}_{s}^{2}$$

③ Huber loss function:

(10) $$L_{\varepsilon }(y,f(x))=\left\{\begin{array}{c} c\left|y-f(x)\right|-c^{2}/2,\left|y-f(x,a)\right|>c\\ {\left|y-f(x)\right|}^{2}/2,\;else \end{array}\right.$$

where *c* is the parameter estimated according to the actual prediction situation.

*ε* insensitive loss function is the most commonly used in SVR. Using this function does not make all samples become support vectors. The number of support vectors can be controlled by the size of *ε* [23].

Different kernel functions correspond to different support vector algorithms. According to Vapnik et al. [16], the kernel function of SVR must first satisfy Mercer’s theorem, that is, if the function k satisfies

(11) $$\int_{\chi \times \chi }k(x,x^{\prime })f(x)f(x^{\prime })dxdx^{\prime }\geq 0,\forall f\in L_{2}(\chi )$$

Then *k*(*x*, *xꞌ*) is a kernel function, which can also be written as *k*(*x*, *xꞌ*) = *φ*(*x*)∙*φ*(*xꞌ*) on feature space.

Here it is assumed that the adopted kernel function satisfies Mercer’s theorem, i.e., it is symmetric and positive semi-definite, so that it can be interpreted as an inner product in some high-dimensional Hilbert space. This ensures the equivalence between nonlinear mapping and kernel representation. If *k*_1_(*x*, *xꞌ*) and *k*_2_(*x*, *xꞌ*) are kernel functions, and *c*_1_, *c*_2_ are constants greater than zero, then *k*(*x*, *xꞌ*) = *c*_1_*k*_1_(*x*, *xꞌ*) + *c*_2_*k*_2_(*x*, *xꞌ*) are also kernel functions. If *k*_1_(*x*, *zꞌ*) is a symmetric function at *χ* × *χ*, then *k*(*x*, *xꞌ*) = ∫*_χ_ k*_1_(*x*, *xꞌ*) *k*_1_(*x*, *zꞌ*) *dz* is also a kernel function.

The Gaussian kernel function satisfies Mercer’s theorem as a commonly used radial basis function. It has high precision in nonlinear fitting and is selected as a basis function for SVR. It is expressed as

(12) $$K(x_{i}\cdot x_{j})=\exp\left(-\frac{{\left\|x_{i}-x_{j}\right\|}^{2}}{2\sigma^{2}}\right)$$

where *σ* is the kernel function’s width parameter, its value dramatically influences the regression accuracy of the SVR.

Based on these classical SVR formulations, this study extends the framework to establish a mapping between mesoscopic parameters of hydraulic concrete and load–CMOD responses. The relationship between load-CMOD and mesoscopic parameters has a high degree of nonlinearity due to the multiphase composition of hydraulic concrete and is difficult to describe with explicit mathematical expressions. Therefore, SVR is proposed to establish the mapping relationship between the mesoscopic parameters of each phase material and the load–CMOD:

(13) $$SVR(X):R^{n}\to r$$

(14) $$U=SVR\left(X\right)$$

(15) $$X=\left\{x_{1},x_{2},\cdots ,x_{n}\right\}$$

where *U* is the CMOD obtained by the mesoscopic numerical simulation, and ***X*** = {*x*_1_, *x*_2_, …, *x_n_*} is the mesoscopic parameters to be identified, such as ***X*** = {*E*, *μ*, *f_t_*, *f_c_*, *k*, *φ*} of mortar, where *E* is the modulus of elasticity, *μ* is Poisson’s ratio, *f_t_* is tensile strength, *f_c_* is compressive strength, *k* is elastic stiffness, and *φ* is shear expansion angle. Equations (13)–(15) are therefore original to this work and represent the nonlinear relationship between multiple mesoscopic parameters and the CMOD response. The number of *SVR*(*x*_1_, *x*_2_, …*x_n_*) is consistent with freedom degrees of different CMODs, that is, the nonlinear relationship between *n* freedom degrees corresponding to *n* mesoscopic parameters to be inverted and the CMODs.

## 3. HGWO Algorithm for Critical Parameters of SVR and Mesoscopic Model

The HGWO has a high optimization ability, so the critical parameters of the SVR will be optimized with the help of the HGWO to obtain a higher performance SVR model and then use it to predict the mesoscopic numerical simulation. The mesoscopic parameter inversion model of hydraulic concrete is also constructed based on HGWO.

### 3.1. Basic Principle of HGWO Algorithms

**Figure 2:**
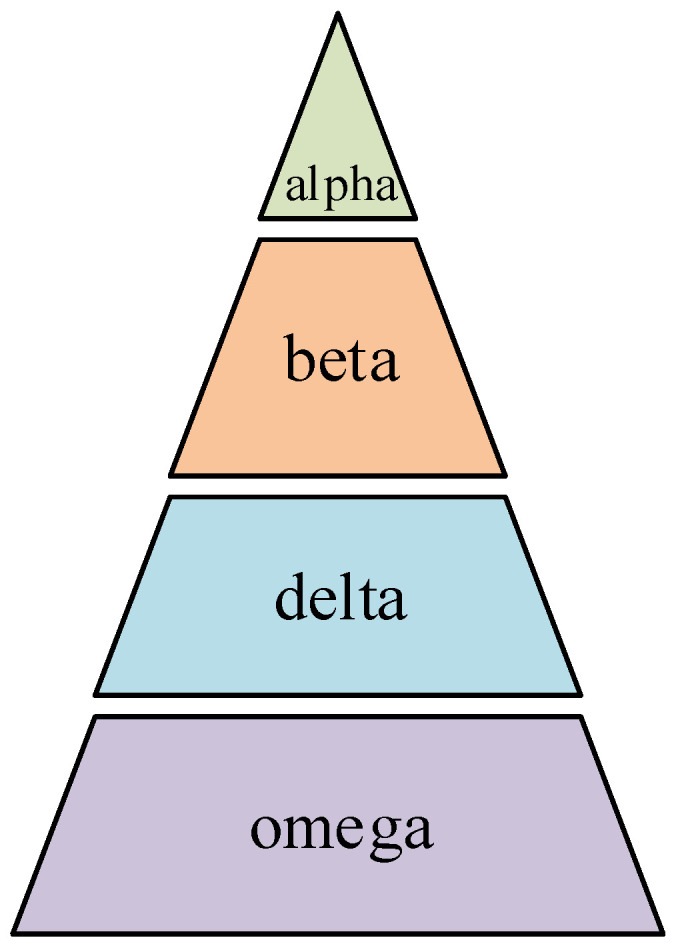
Population structure of grey wolves.

The Grey Wolf Optimization (GWO) algorithm simulates the group predation behavior of grey wolves. It achieves the optimization purpose based on the wolf group tracking approach, hunting, encircling, and attacking prey [24]. As shown in Figure 2, wolves have a strict hierarchy of social domination. The group’s leader is the alpha wolf, who decides everything in the population, and all its decisions are transmitted to the entire population and obeyed. Beta’s status is second only to alpha. It assists alpha in making decisions and gives alpha feedback. It is also the successor of alpha. In the third class is the delta wolf, whose main task is finding prey, acting as a scout to keep the population safe, and obeying alpha and beta’s orders. At the bottom are omega wolves, who must obey all commands of other wolves. The optimization process of the GWO algorithm is as follows: a group of grey wolves is randomly generated in the search domain, and the grey wolves in the wolf pack are ranked from highest to lowest as alpha, beta, delta, and omega. Alpha, beta, and delta evaluate the position of the prey. They are jointly responsible for specifying the movement direction of the omega wolf, realizing the encirclement attack on the prey, and ultimately capturing the prey.

The grey wolf algorithm has a fast convergence speed and high optimization accuracy, but it easily falls into local optimization for some complex problems. Differential evolution (DE) has global solid search capabilities but is sensitive to parameters and weak local search capabilities. In order to give full play to their respective advantages and compensate for the existing shortcomings, Zhu et al. [25] integrated DE into GWO to obtain a new hybrid grey wolf optimization (HGWO) algorithm. In this study, we adapt and extend this concept to construct the HGWO–SVR inversion framework. The implementation steps are expressed mathematically in Equations (16)–(24), which represent the original contribution of this work in the context of parameter inversion.

(1) The initialization parameters mainly include basic parameters such as population size *N*, maximum iteration number *t*_max_, crossover probability *C_R_*, scale factor *F* range, and randomly generate three initial populations of the same size by Equation (16):

(16) X(0)pk=Xplow+rand(0,1)×(Xpup−Xplow)

where *X_p_^k^*(0) is the *p*-th dimension value of the *k*-th individual in the initial population; *rand*(0, 1) represents the random number generated in the range of [0, 1]; *X_p_^up^* and *X_p_^low^* are the upper and lower bounds of the *p*-th dimension value.

Denote an initial population by POP, which can be defined by the following formula:

(17) $$POP=\left\{X^{1},X^{2},\cdots ,X^{psize}\right\}$$

where *psize* is the population size and *k* is the individual number, *k* = 1, 2, 3…, *psize*. Each individual can be represented by:

(18) $$X^{k}=\left(X_{1}^{k},X_{2}^{k},\cdots X_{p}^{k},\cdots X_{d}^{k}\right)$$

where *k* = 1, 2, 3…, *d* and *k* = 1, 2, 3…, *psize*.

Sort the parent population in non-decreasing order to find the parent individuals ranked first, second, and third in the population, which are called *α*, *β*, and *δ*, respectively.

(2) The DE mutation operation is performed on the population of individuals. The realization process is to generate intermediates and perform competitive selection operations to form parent, offspring, and mutant population individuals by Formula (19).

(19) $$V_{p}^{k}(t+1)=X_{p}^{r1}(t)+F\times (X_{p}^{r2}(t)-X_{p}^{r3}(t)),r1\neq r2\neq r3\neq k$$

where *t* represents the current number of iterations, and *r*1, *r*2, and *r*3 represent three different random numbers.

(3) The fitness value of each grey wolf in the population was calculated and sorted according to the fitness value. The optimal top 3 individual positions were selected as *X_α_*, *X_β_*_,_ and *X_δ_*_,_ respectively.

(4) Calculate the distances between other grey wolf and the optimal *X_α_*, *X_β_* and *X_δ_* in the population according to Equation (20), and update the current position of each grey wolf individual according to Equations (21) and (22).

(20) $$D_{\alpha }=\left|C_{1}X_{\alpha }(t)-X(t)\right|;D_{\beta }=\left|C_{2}X_{\beta }(t)-X(t)\right|;D_{\delta }=\left|C_{3}X_{\delta }(t)-X(t)\right|$$

(21) $$X_{1}(t+1)=X_{\alpha }(t)-A_{1}D_{\alpha },X_{2}(t+1)=X_{\beta }(t)-A_{2}D_{\beta },X_{3}(t+1)=X_{\delta }(t)-A_{3}D_{\delta }$$

(22) $$X(t+1)=\frac{X_{1}+X_{2}+X_{3}}{3}$$

where *t* is the current number of iterations; *A* is the convergence factor; *C* is the swing factor; *D* is the distance between the grey wolf and the optimal individual position *X_α_*, *X_β_*_,_ and *X_δ_*; *X*(*t*) is the grey wolf position for the *t*-th iteration; *a* is a variable decrease linearly from 2 to 0 as the iterations number increases; and *r*_1_ and *r*_2_ are random numbers in the range of [0, 1].

(5) According to Formula (23), the population individuals are crossed and selected, excellent components are retained, new offspring are generated, and the fitness value of individuals is calculated.

(23) $$U_{p}^{k}(t+1)=\left\{\begin{array}{l} V_{p}^{k}(t+1),\;if\;rand(0,1)\leq P_{CR}\;or\;p=p_{rand}\\ X_{p}^{k}(t),\;if\;rand(0,1)>P_{CR}\;or\;p\neq p_{rand} \end{array}\right.$$

where *U*(*t* + 1) indicates a new variant of *V*(*t* + 1) after crossover; *P_CR_* is a constant that represents a specific crossover probability; and *p_rand_* denotes random dimensions.

DE uses greedy criteria to determine whether to retain new variant individuals *U*(*t* + 1) into the next generation. The action can be expressed as

(24) $$X(t+1)=\left\{\begin{array}{c} U(t+1),\;if\;f(U(t+1))\leq f(X(t+1))\\ X(t),\;if\;f(U(t+1))>f(X(t+1)) \end{array}\right.$$

where *f* is the fitness function.

(6) Update parameters *a*, *A* and *C*.

(7) Determine whether the maximum number of iterations *t*_max_ has been reached. If so, stop the iteration and output the current optimal solution. Otherwise, return to step (3) to continue the iteration.

### 3.2. HGWO for Key Parameters of SVR and Mesoscopic Model

#### 3.2.1. Implementation Process of HGWO Algorithm for Key Parameters of SVR

As one of the hyperparameters of the SVM model, penalty factor *C* is one of the key parameters to ensure performance. The larger the value of *C*, the closer the penalized slack variable is to 0, which means that the cost of the wrong prediction is higher, so the accuracy of the training set test is high, but the generalization ability is weak. The smaller the value of *C*, the lower cost of false prediction, the fault tolerance is allowed, the wrong prediction point is regarded as a noise point, and the generalization ability is strong. Even in the case of linear separability, if the value of *C* is too small, misprediction may occur. In the case of linear inseparability, if the value of *C* is tremendous, the training process may fail to converge. The default value of the *C* is 1.0, and filtering step by step from an extensive range is needed until finding the most suitable *C*.

Insensitivity coefficient *g* is a parameter that comes with selecting the Gaussian radial basis function as kernel function, which implicitly determines the data distribution after mapping to a new feature space. The larger the *g*, the fewer support vectors. The smaller the *g* value, the more support vectors. The number of support vectors will affect the training and prediction speed. The relationship between *σ* and *g* in the RBF kernel function is as follows:

(25) $$k(x,z)=\exp\left(-\frac{d{\left(x,z\right)}^{2}}{2\sigma^{2}}\right)=\exp(-g\cdot d{\left(x,z\right)}^{2})\Rightarrow g=\frac{1}{2\sigma^{2}}$$

The value of *g* will affect the range of Gaussian action corresponding to each support vector, thus affecting the generalization performance. If the *g* value is too large, the *σ* value will be minimal, which will make the Gaussian distribution high and thin, thus limiting the affecting scope of the support vector samples. The prediction effect for unknown samples will be inferior. Theoretically, if *σ* is infinitely tiny, the SVR of the Gaussian kernel can fit any nonlinear data, which can easily cause over-fitting, and the prediction accuracy is not high. However, the smoothing effect will be too significant if the value of *g* is too tiny, and higher accuracy cannot be obtained on the training set, which will also affect the prediction accuracy.

**Figure 3:**
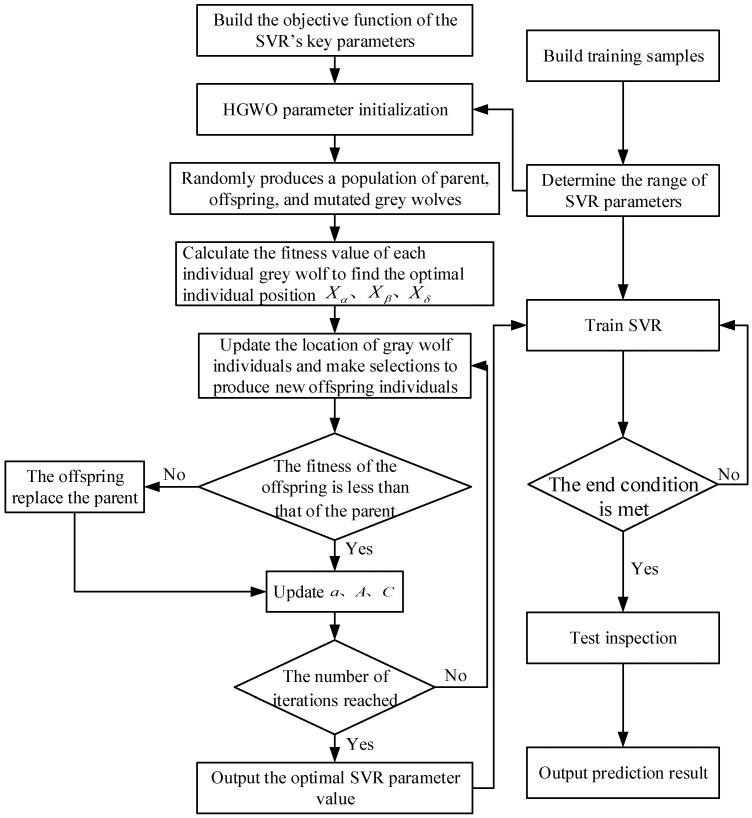
Flow chart of HGWO SVR parameter algorithm.

Therefore, the values of *C* and *g* will considerably impact the SVR model’s performance, which becomes a crucial factor in obtaining an ideal SVR model. To improve the grid’s generalization ability and prediction accuracy, the HGWO algorithm was introduced into the sample training process of SVR, and the original random selection of SVR parameters evolved into an efficient, reliable, and evidence-based selection. The process of the HGWO algorithm searching for SVR parameters is shown in Figure 3, and the specific steps are as follows.

(1) Objective function establishment

The objective function value is the optimization basis for the HGWO algorithm, which maps individual space to real topological space. Take the minimum squared error between the SVR prediction value and the mesoscopic numerical result of the CMOD as the basis for selecting the optimal *C* and *g* values. Therefore, the objective function can be established as

(26) $$F(X)={\left\{\frac{1}{k}{\sum_{i=1}^{m}\left[SVR_{i}(X)-U_{i}\right]}^{2}\right\}}^{1/2}$$

where *X* = {*C*, *g*} is a set of parameters to be optimized; *SVR_i_*(***X***) is the predicted value of the CMOD; *U_i_* is the mesoscopic numerical analysis result of CMOD under TPB. *m* is the total data obtained by mesoscopic numerical simulation.

The optimization calculation of SVR parameters solves the equation’s objective function (26), looking for an appropriate set of *C* and *g* values to minimize the corresponding objective function value.

(2) Initialize the parameters of the HGWO algorithm

Set the main parameters of the HGWO algorithm (such as population size, zoom factor range, crossover probability, population iteration times, etc.). The value range of the optimization variables *C* and *g* are set according to the specific problem, which is convenient for the quick search of the mixed grey wolf.

(3) Algorithm iteration conditions

Based on the set initial parameters of the HGWO algorithm, the fitness function value is calculated, and the position and moving direction of the grey wolf are adjusted. The iteration is ended when the optimization accuracy criterion or the number of iterations is reached. The current optimal result is returned as the optimal *C* and *g*.

In summary, when applied to practical problems, the HGWO algorithm first determines the training sample parameters of the SVR and finds the best *C* and *g* values. Then, they are substituted into the SVR, and we establish the model and perform simulation learning and comparative analysis of prediction samples.

The following performance evaluation indexes evaluate the prediction accuracy of the constructed SVR model:

(1) Squared Correlation Coefficient [26], *R*^2^

(27) $$R^{2}=\frac{{\left(\sum_{i=1}^{n}(y_{i}-\bar{y})({\overset{⌢}{y}}_{i}-{\bar{y}}^{\prime }\right)}^{2}}{\sum_{i=1}^{n}{\left(y_{i}-\bar{y}\right)}^{2}\sum_{i=1}^{n}{\left({\hat{y}}_{i}-{\bar{y}}^{\prime }\right)}^{2}}$$

where *y_i_* is the measured value; ${\hat{y}}_{i}$ is the prediction value; $\bar{y}$ is the mean value of the original measured value; ${\bar{y}}^{\prime }$ is the mean value of the predicted value; and *n* is the number of samples.

This indicator is widely used to measure the model fitting effect. The closer the value is to 1, the better the prediction effect.

(2) Mean Absolute Error [27], *MAE*

(28) $$MAE=\frac{1}{n}\sum_{i=1}^{n}\left|y_{i}-{\hat{y}}_{i}\right|$$

This indicator is the average of the absolute error and reflects the situation of the prediction error.

(3) Mean Absolute Percentage Error [28], *MAPE*

(29) $$MAPE=\frac{1}{n}\sum_{i=1}^{n}\left|\frac{y_{i}-{\hat{y}}_{i}}{y_{i}}\right|$$

This indicator is the average value of the relative error, which reflects the size of the error relative to the measured value. However, it cannot reflect the size of the absolute error.

(4) Mean Squared Error, *MSE*

(30) $$MSE=\frac{1}{n}\sum_{i=1}^{n}(y_{i}-{\hat{y}}_{i}{)}^{2}$$

This indicator is the mean of the squared difference between the predicted value and the measured value, and the smaller the MSE, the better the prediction.

#### 3.2.2. HGWO Algorithm for Key Mesoscopic Parameters of Hydraulic Concrete

The mesoscopic parameters inversion of hydraulic concrete based on the numerical simulation calculation and physical test data is essentially equivalent to the following problems:

(31) $$\begin{array}{l} \mathrm{minimize}:f(x)\\ \mathrm{Subject}\;\mathrm{to}:K(x)u=F\\ x\in D_{x} \end{array}$$

where *f*(***x***) is the optimized objective function; ***x*** = {*x*_1_, *x*_2_, …, *x_m_*} is the mesoscale parameter to be identified; and *D_x_* is the feasible region of the parameter.

The objective optimization function of mesoscale parameter inversion is

(32) $$f(x)={\left\{\frac{1}{k}\sum_{i=1}^{n}{\left(SVR_{i}(x)-u_{i}\right)}^{2}\right\}}^{1/2}$$

where *SVR_i_*(***x***) is the predicted value of CMOD obtained by the SVR model; ***x*** = {*x*_1_, *x*_2_, …, *x_m_*} is the mesoscale parameter to be identified; *u_i_* is the measured value of CMOD corresponding to the *i*-th research point; and *n* is the number of study points selected from the experimental results (for example, in the case of mesoscale parameter inversion, this value represents the number of CMODs points extracted corresponding to several specific time).

**Figure 4:**
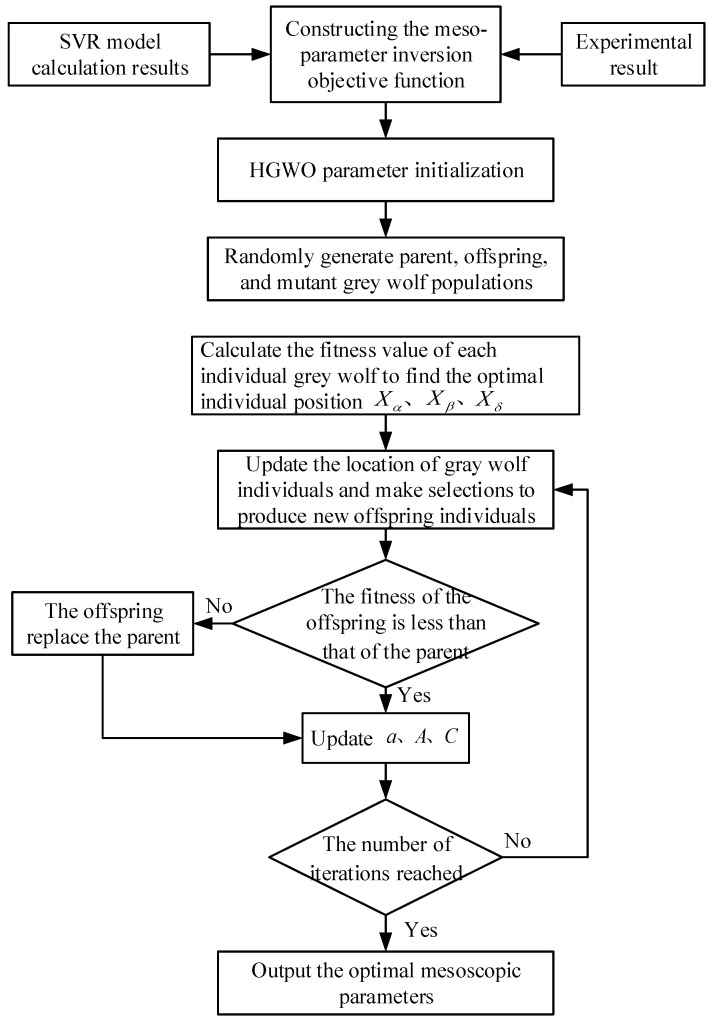
Flowchart of HGWO parameter identification algorithm for mesoscale model.

The basic process of the mesoscale parameter inversion algorithm based on the HGWO algorithm is similar to the basic process of the SVR parameter optimization. The most significant difference is the objective function. The objective function of the HGWO algorithm for parameter optimization of the SVR is established based on the relationship between the results of the mesoscale numerical simulation and the predicted value of the SVR model. However, the objective function of the mesoscale parameter inversion is based on the relationship between the experimentally measured value and the SVR model’s predicted value. The mesoscale parameter inversion process based on the HGWO algorithm is shown in Figure 4.

## 4. The Realization Process of 3D Mesoscopic Model Parameter Inversion of Hydraulic Concrete

**Figure 5:**
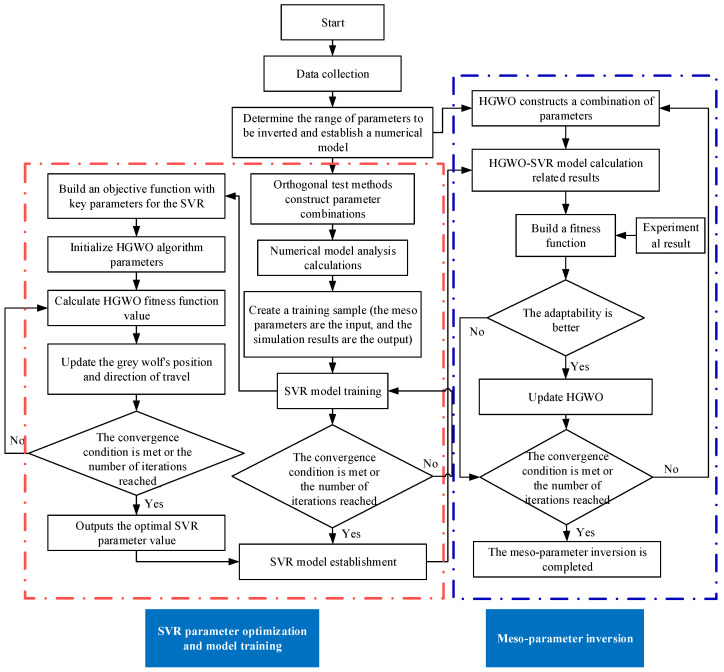
Parameter inversion flow chart.

The specific implementation process of hydraulic concrete parameter inversion is shown in Figure 5. It mainly includes two parts: ① Optimize parameters *C* and *g* of SVR by the HGWO algorithm. The specific implementation steps have been described in detail in Section 3.2.1; ② Search for the mesoscopic parameters closest to the measured data and the results calculated by the trained SVR through the HGWO algorithm. The two parts are built and combined in an orderly manner. The specific steps are as follows:

(1) Determine the number of parameters to be identified and the corresponding value intervals. Select representative and decisive key parameters for specific problems, the scope of which can be obtained by experiments, trial calculations, experience, analogies, etc.

(2) Build sample parameters. Generally, it can be constructed in a variety of ways. If the number of parameters and the division levels are both large, the Taguchi methodology, uniform experimental method, or random sampling method can be used. If the number of parameters and levels is within the controllable range, then a full test method can be selected.

(3) Parameter normalization. It should be noted that the dataset composed of mesoscale parameters and simulation results need to be normalized before being used for SVR. The following equation is used for the data normalization.

(33) $$f:x\to y=\frac{2(x-x_{\min})}{x_{\max}-x_{\min}}+1$$

where *x* = (*x*_1_, *x*_2_, …, *x_n_*) is the raw data; *y* = (*y*_1_, *y*_2_, …, *y_n_*) is the result of the normalization of the corresponding *x*, and *y_i_* ∈ *R*, *i* = 1, 2, …, *n*, min(*x*) is the minimum value of sample *x*; max(*x*) is the maximum value of sample *x*.

(4) Using the above samples as training data, the optimal SVR parameters (*C*, *g*) are achieved by the process detailed in Section 3.2.1 under the training sample. In addition, these samples should be used as a set to test the regression effect of the trained SVR model. The trained SVR model is the established nonlinear mapping relationship between the input parameters to be inversed and the output results. It can also be regarded as a regression SVR with a prediction function obtained by repeated learning of the training data.

(5) The number of mesoscopic parameters to be identified and the corresponding value range must be determined before the inversion. The Taguchi methodology is used to construct the parameter combinations to be identified. Then the trained SVR model is used to predict the corresponding value (It is worth noting that the trained SVR prediction results replace the results of the mesoscale numerical simulation). As a result, the prediction result and the measured data are used to construct a fitness function, as shown in Equation (32).

(6) The parameter inversion process is completed when the adaptability value achieves the expected accuracy or the iterations reach the maximum number. Then we output the inversed parameters.

## 5. Case Study

The TPB beam specimen T2 test results of the tertiary hydraulic concrete in the author’s published paper are selected [29], and the mortar’s mesoscale parameters are inversely analyzed by the proposed method.

### 5.1. Experiment Design Based on the Taguchi Method

According to research [5], the damage plasticity constitutive relation is suitable for mortar. It includes multiple parameters such as elastic modulus, tensile strength, compressive strength, Poisson’s ratio, density, and critical strain. We choose the first three parameters to inverse. The Poisson’s ratio, density, and critical strain of mortar are determined based on previous publications [6], and the unknown parameters’ value range is determined through the relevant experimental results shown in Table 1.

Based on some results in the previous trial calculations, each mesoscopic parameter is assigned five factor levels [30], as listed in Table 2. If all parameter levels in Table 2 are entirely combined, there are 125 groups of parameter combinations. To reduce computational cost, the Taguchi orthogonal array method [31] was adopted. In this study, the number of factors is 3 (elastic modulus, tensile strength, and compressive strength), and each factor has 5 levels. The corresponding Taguchi L_25_(5^6^) orthogonal array is shown in Table 3. As seen from the table, only 25 sets of tests are needed, which can significantly improve the calculation efficiency.

In addition to reducing the number of simulations, the Taguchi method provides a systematic and efficient approach for exploring the influence of multiple factors on model outputs. By arranging parameters according to an orthogonal array, the method ensures balanced coverage of the design space and allows the main effects of each factor to be quantified with a limited number of experiments. This feature is particularly suitable for computationally intensive mesoscopic simulations of hydraulic concrete. However, it should be noted that the Taguchi method primarily focuses on main effects and may not fully capture higher-order interactions between factors. Therefore, while it greatly improves efficiency, complementary validation or sensitivity analysis may still be necessary for cases where factor interactions are expected to play a dominant role.

### 5.2. Numerical Model Establishment for Mesoscopic Analysis

The proposed HGWO model has been applied to inverse the tertiary hydraulic concrete’s mortar mesoscopic parameters. Firstly, a mesoscopic numerical model is established according to the composition of the tertiary TPB beam. The mesoscopic parameter combination scheme obtained in the previous section is used in the numerical model calculation, and the corresponding P-CMOD is obtained.

Spherical aggregate is used in the tertiary hydraulic concrete. The aggregate sieving ratio and mix proportions used in this study are consistent with those reported in our previous work [32]. For detailed parameter values, please refer to Tables 2 and 3 in ref. [32]. The aggregate volume fraction is 40%, slightly lower than the aggregate content of the actual specimen. It can be learned from Grassl [29] that the influence of aggregate content on the simulation results is limited. From the research of Chen [33], it can be known that the thickness of ITZ in solid concrete is generally between 50 μm and 100 μm. Inserting a zero-thickness cohesive element between aggregate and mortar matrix can not only characterize the cracking process of the ITZ but also make the ITZ thickness close to the actual thickness, reducing the simulation error caused by the ITZ thickness difference [31,33]. To ensure a better mesh and iterate efficiently, we characterize ITZ by inserting zero-thickness cohesive elements and assigning cohesive elements constitutive.

In this modeling strategy, the mortar and ITZ are represented using a traction–separation cohesive zone model (CZM), while the aggregates are assumed to be linear elastic. All solid phases (mortar matrix and aggregates) are modeled with 3D eight-node linear brick elements (C3D8 in ABAQUS), whereas ITZ is discretized using zero-thickness cohesive elements (COH3D8). This selection allows explicit representation of crack initiation and propagation along mortar–aggregate interfaces. A relatively fine mesh (typical element size ≈ 1.5 mm) was used around the prefabricated notch to capture CMOD evolution accurately, and a coarser mesh was applied in non-critical regions to reduce computational cost.

**Figure 6:**
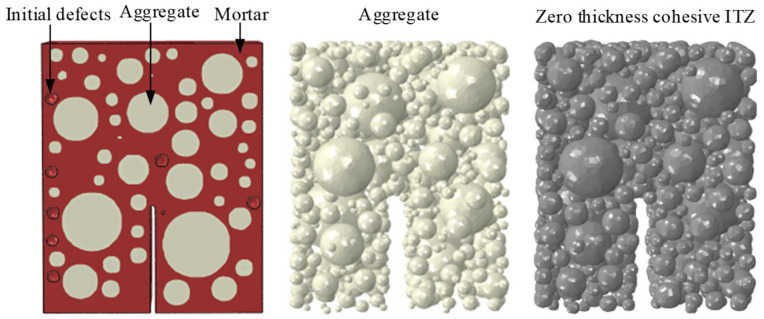
The material components in the mesoscopic model of the TPB beam.

In the study of Wang [34], it was found that the initial defect dramatically influences the specimen’s overall strength and failure mode. The initial defects are produced due to insufficient vibration or maintenance, which is the weakest zone in the concrete specimen. Therefore, it is more reasonable to introduce initial defects into the numerical model. This paper’s TPB models of hydraulic concrete all contain 1% initial spherical defects. Liu pointed out that in common strength concrete under a low loading rate, the aggregates often do not break, and always hinder the development of cracks [35]. So in this simulation, the aggregate is regarded as a linear elastic material. The TPB mesoscopic model was established by the author’s published paper method [6], and a zero-thickness cohesive element was inserted between the mortar and the aggregate through self-programming. The final established model is shown in Figure 6.

**Figure 7:**
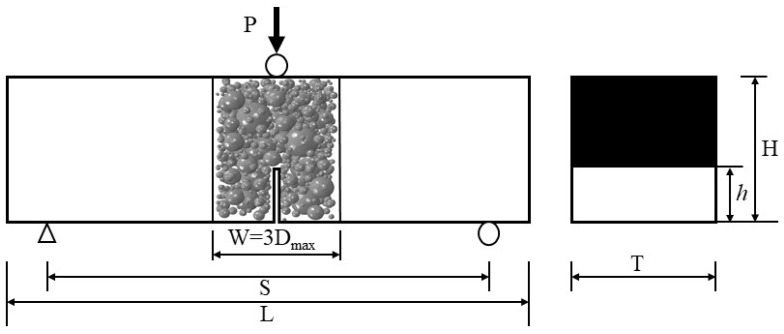
Schematic diagram of TPB numerical simulation of hydraulic concrete.

As shown in Figure 7, the displacement load is applied to the specimen through the rigid body in the center. Simply support constraints are set on the support positions at both ends of the specimen. The left end support constrains the displacements in the *x*, *y*, and *z* directions, and the right end support constrains the displacements in the *x*, *y* directions. In order to eliminate the rate effect in the loading process, the numerical simulation adopts the 0.001 mm/s quasi-static loading rate.

In the process of 3D mesoscopic simulation, the mortar parameters used are shown in Table 3. The aggregate, homogeneous body, and ITZ model parameters are determined based on previous works [36,37]. The mesoscopic model parameters of each phase material are shown in Table 4.

The simulation results are extracted by the self-compiled Python program, implemented in Python 3.9. The load P in the load–CMOD curve is the interaction force between the rigid body and the specimen. The CMOD is obtained by extracting the mean relative displacement of nodes at both ends of the prefabricated crack.

### 5.3. Optimization of Key Parameters of SVR and Analysis of Results

(1) Comparison of optimization ability before and after improvement of GWO algorithm

Taking each set of mesoscopic parameters above and the CMOD corresponding to time obtained by numerical calculation as a set of samples, 25 sets of data were obtained. Then the DE-SVR, GWO-SVR, and HGWO-SVR algorithms were used for SVR parameter optimization. The convergence effect and prediction accuracy of the three intelligent algorithms are compared. RBF kernel function is used for all models. The initialization parameters of GWO-SVR are set as follows: the population size is 30, the number of population iterations is 200, the *ε* value is 0.01, the penalty factor *C* and the insensitivity coefficient *g* are in the range of [0.01, 100]. For DE-SVR algorithms: population size is 30, number of iterations is 200, lower bound of the scale factor is 0.2, upper bound of the scale factor is 0.8, the cross probability is 0.2, ε value is 0.01, penalty factor *C* and insensitive coefficient *g* are in the range of [0.01,100]. The HGWO-SVR algorithm has the same initialization parameter settings as the DE-SVR.

**Figure 8:**
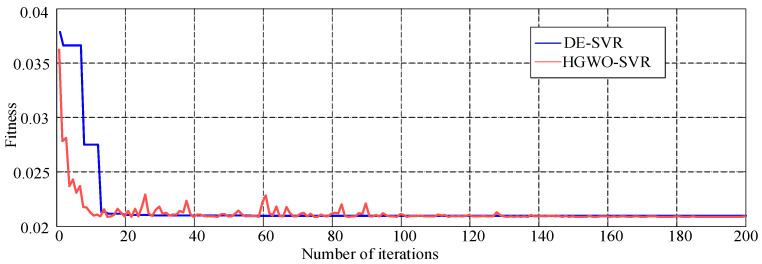
Comparison of convergence effect between DE-SVR and HGWO-SVR.

The convergence process of DE-SVR and HGWO-SVR algorithms is shown in Figure 8. As shown in Figure 8, the optimal adaptability of the HGWO and DE algorithm is close when the operation converges. However, the DE algorithm falls into two local optimizations, while HGWO does not fall into the local optimality. Therefore, the GWO algorithm can keep the DE algorithm from falling into local optimization.

The SVR model was optimized ten times with the improved and unimproved GWO algorithm. The mean calculation time, convergence iteration times, and model prediction accuracy are shown in Table 5. It can be seen from Table 5 that the HGWO-SVR algorithm has no advantage in reducing the number of iterations and shortening the operation time compared with the GWO-SVR. However, the HGWO-SVR model has apparent advantages in prediction accuracy. The MAE, MSE, and MAPE of the HGWO-SVR model are all smaller than the GWO-SVR model. The prediction accuracy of HGWO-SVR is 94.4%, which is 4.8% higher than the GWO-SVR model (89.6%). The prediction accuracy and the number of iterations of the DE-SVR are close to HGWO-SVR. However, the running time of the DE-SVR model is significantly longer than HGWO-SVR. Therefore, combining the GWO algorithm can improve DE’s computational efficiency. It can be seen that the model prediction effect is significantly improved after the DE algorithm is integrated into the GWO-SVR. Although the number of iterations and the optimization speed are slowed down, its optimization ability is stronger than the GWO algorithm, so the DE algorithm can improve the model’s prediction accuracy. Compared with the DE-SVR algorithm, after the GWO algorithm is integrated into the DE-SVR, the prediction accuracy has almost no changed. However, the optimization speed is significantly faster, and the running time is significantly reduced. The GWO algorithm can speed up the optimization and reduce the running time.

Using the above HGWO-SVR model, after a specific iterative calculation, the optimal parameters in the SVR searched by HGWO are shown in Table 6.

### 5.4. Mesoscopic Parameters of Hydraulic Concrete Inversion and Results Analysis

**Figure 9:**
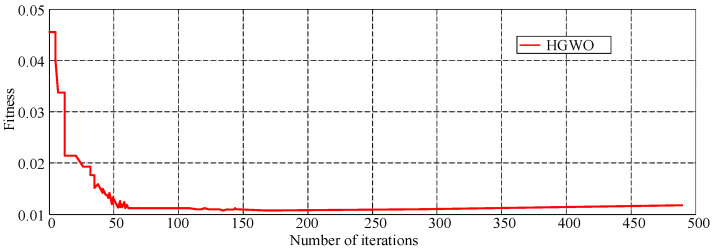
Convergence rate of HGWO inversion of mesoscale parameters.

The SVR model is established after obtaining the optimal parameter through the HGWO algorithm. Then the objective function is established by SVR prediction and TPB experiment’s CMOD (using the test result of T2 specimen in the literature [32]) corresponding to 5 loading points (0.5 P of pre-peak, 0.8 P of pre-peak, P, 0.8 P of post-peak, 0.5 P of post-peak, P is the peak load). The initial parameters of the HGWO are set as follows: the population size is 30, the number of iterations is 500, the lower and upper bound of the scaling factor are 0.2 and 0.8, and the crossover probability is 0.2, and the *ε* value is 0.01. The algorithm is iterated until the fitness reaches the optimum, and the convergence process is shown in Figure 9. The optimization mesoscopic parameters results of 60 iterations are the best, as shown in Table 7.

### 5.5. TPB Numerical Simulation and Result Analysis of Inversed Mesoscopic Parameters

**Figure 10:**
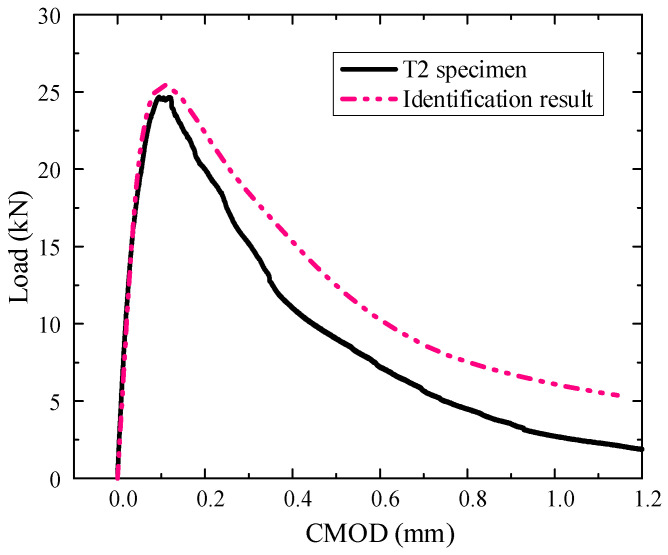
The P-CMOD curve obtained from the simulation results of inversion parameters.

The TPB mesoscopic simulation of the hydraulic concrete was carried out based on the inversed parameters. The obtained results were compared with the physical test results, as shown in Figure 10. It can be seen from the figure that the CMOD based on the inversed mesoscopic parameters is well consistent with the test result data. The rising slope of the P-CMOD curve is relatively close, indicating that the inversed elastic modulus of the mortar is very close to the actual hydraulic concrete. The peak loads obtained based on the inversion results are close to the test results, indicating that the inversed mortar tensile strength is also close to the practical specimen. However, the development of the post-peak curve has a distinct difference which may be caused by the different distribution of aggregates and defects. Since the established mesoscopic model is highly simplified, thus caused the difference in the post-peak curve. Overall, the method proposed can efficiently inverse the mesoscopic parameters of the hydraulic concrete.

## 6. Conclusions

HGWO and SVR were introduced to inverse the parameters of the 3D mesoscopic mechanical analysis model of hydraulic concrete with the full support of physical experiments and 3D mesoscopic numerical simulations. The main conclusions are as follows:

(1) An SVR model was established to capture the relationship between the mesoscopic parameters and the macroscopic mechanical response of hydraulic concrete. Furthermore, an HGWO method was proposed to optimize the core parameters of SVR (penalty factor *C* and insensitivity coefficient *g*). Application examples demonstrate that optimization significantly improves the accuracy and convergence speed of the SVR model.

(2) The proposed SVR prediction model was designed to replace repetitive and computationally expensive 3D mesoscopic numerical calculations. By combining physical experiments, an inversion objective function of hydraulic concrete parameters was formulated. The integration of SVR’s high-precision forecasting capability with HGWO’s efficient search ability greatly enhances both the accuracy and efficiency of parameter inversion.

(3) Taking the hydraulic concrete beam TPB test as an example, the proposed method successfully inverted the mesoscopic mechanical parameters of mortar, and the inversion results were applied to TPB numerical simulations. The predicted CMOD values exhibited both magnitude and trend consistency with the experimental results, confirming the feasibility of the method and the reliability of the identified parameters.

Despite these promising results, the proposed method still has certain limitations. The inversion accuracy may depend on the representativeness of the training database and the selected mesoscopic model assumptions. In addition, the current study mainly focused on concrete beam tests; further validation on other structural configurations and loading conditions is required. Future work should also consider coupling with multi-scale models and extending the algorithm to handle environmental factors such as temperature and moisture variations.

## Figures and Tables

**Table 1:** The mesoscopic parameter’s value and range of mortar.

Mesoscopic Parameters	Poisson’s Ratio	Density/ kg/m^3^	Critical Strain	Elastic Modulus/GPa	Tensile Strength/MPa	Compressive Strength/MPa
Range	0.2	2000	3.55 × 10^−3^	[20, 40]	[1.5, 3.5]	[40, 60]

**Table 2:** Selection levels of mesoscale parameters.

Factor Level	Mesoscopic Parameters of Mortar		
	Elastic Modulus/GPa	Tensile Strength/MPa	Compressive Strength/MPa
1	20	1.5	40
2	25	2.0	45
3	30	2.5	50
4	35	3.0	55
5	40	3.5	60

**Table 3:** Calculation scheme of mesoscale parameters based on Taguchi design.

Scheme	Mesoscopic Parameters of Mortar		
	Elastic Modulus/GPa	Tensile Strength/MPa	Compressive Strength/MPa
1	20	1.5	40
2	25	2.0	40
3	30	2.5	40
4	35	3.0	40
5	40	3.5	40
6	40	3.0	45
7	35	2.5	45
8	30	2.0	45
9	25	1.5	45
10	20	3.5	45
11	20	3.0	50
12	25	2.5	50
13	30	3.5	50
14	35	2.0	50
15	40	2.5	50
16	40	1.5	55
17	35	2.0	55
18	30	3.0	55
19	25	3.5	55
20	20	2.5	55
21	20	2.0	60
22	25	3.0	60
23	30	1.5	60
24	35	3.5	60
25	40	2.5	60

**Table 4:** Material parameters of meso-scale hydraulic concrete model.

Composition	Elastic Modulus (GPa)	Poisson’s Ratio	Density (kg/m^3^)	Elastic Stiffness (MPa/mm)	Cohesion Strength (MPa)	Fracture Energy (N/mm)
Homogenous body	28	0.2	2400	--	--	--
Aggregate	40	0.2	2600	--	--	--
ITZ	--	--	2000	10^5^	3.5	0.03

**Table 5:** Prediction effect analysis of GWO-SVR, DE-SVR, and HGWO-SVR models.

Index	The Optimal Number of Iterations	MAE	MSE	MAPE	R^2^	Computation Time (s)
GWO-SVR	349	1.533	3.976	0.048	0.896	0.38
DE-SVR	668	1.219	2.096	0.041	0.944	13,360.87
HGWO-SVR	724	1.220	2.101	0.041	0.944	789.53

**Table 6:** Optimized SVR parameters.

Parameters	Value Range	Initial Value	Optimization Value
C	[0.01, 100]	10	99.85
g	[0.01, 100]	10	0.1089

**Table 7:** Mesoscale parameters of mortar obtained by inversion.

Mesoscopic Parameters	Elastic Modulus/GPa	Tensile Strength/MPa	Compressive Strength/MPa
Identification result	27.54	2.32	47.52

## Data Availability

The original contributions presented in this study are included in the article. Further inquiries can be directed to the corresponding author.
